# Susceptibility to deltamethrin of wild and domestic populations of *Triatoma infestans* of the Gran Chaco and the Inter-Andean Valleys of Bolivia

**DOI:** 10.1186/s13071-014-0497-3

**Published:** 2014-11-14

**Authors:** Marinely Bustamante Gomez, Grasielle Caldas Pessoa D’Avila, Ana Lineth Garcia Orellana, Mirko Rojas Cortez, Aline Cristine Luiz Rosa, François Noireau, Liléia Gonçalves Diotaiuti

**Affiliations:** Laboratório de Triatomíneos e Epidemiologia da Doença de Chagas, Centro de Pesquisas René Rachou - FIOCRUZ Minas, Belo Horizonte, Brazil; Instituto de Investigaciones Biomédicas – Facultad de Medicina, Universidad Mayor de San Simón, Cochabamba, Bolivia; CEADES Salud y Medio Ambiente, Cochabamba, Bolivia; Institute de Recherche pour le Developemente (IRD), La Paz, Bolivia

**Keywords:** *Triatoma infestans*, Control, Bolivia, Insecticide resistance, Deltamethrin

## Abstract

**Background:**

The persistence of *Triatoma infestans* and the continuous transmission of *Trypanosoma cruzi* in the Inter-Andean Valleys and in the Gran Chaco of Bolivia are of great significance. Coincidentally, it is in these regions the reach of the vector control strategies is limited, and reports of *T. infestans* resistance to insecticides, including in wild populations, have been issued. This study aims to characterize the susceptibility to deltamethrin of wild and domestic populations of *T. infestans* from Bolivia, in order to better understand the extent of this relevant problem.

**Methods:**

Susceptibility to deltamethrin was assessed in nine, wild and domestic, populations of *T. infestans* from the Gran Chaco and the Inter-Andean Valleys of Bolivia. Serial dilutions of deltamethrin in acetone (0.2 μL) were topically applied in first instar nymphs (F1, five days old, fasting, weight 1.2 ± 0.2 mg). Dose response results were analyzed with PROBIT version 2, determining the lethal doses, slope and resistance ratios (RR). Qualitative tests were also performed.

**Results:**

Three wild *T. infestans* dark morph samples of Chaco from the Santa Cruz Department were susceptible to deltamethrin with RR_50_ of <2, and 100% mortality to the diagnostic dose (DD); however, two domestic populations from the same region were less susceptible than the susceptibility reference lineage (RR_50_ of 4.21 and 5.04 respectively and 93% DD). The domestic population of Villa Montes from the Chaco of the Tarija Department presented high levels of resistance (RR_50_ of 129.12 and 0% DD). Moreover, the domestic populations from the Valleys of the Cochabamba Department presented resistance (RR_50_ of 8.49 and 62% DD), the wild populations were less susceptible than SRL and *T. infestans* dark morph populations (RR_50_ < 5).

**Conclusion:**

The elimination of *T. infestans* with pyrethroid insecticides in Brazil, Uruguay, Chile, and its drastic reduction in large parts of Paraguay and Argentina, clearly indicates that pyrethroid resistance was very uncommon in non-Andean regions. The pyrethroid susceptibility of non-Andean *T. infestans* dark morph population, and the resistance towards it, of Andean *T. infestans* wild and domestic populations, indicates that the Andean populations from Bolivia are less susceptible.

## Background

*Triatoma infestans* (Hemiptera, Reduviidae) is the main vector of *Trypanosoma cruzi* (Trypanosomatidae), pathogenic agent of Chagas disease in the countries of the Southern Cone of Latin America [1,2]. Traditionally, the vector control programs focus on the interruption of transmission cycles using insecticides with residual action, especially pyrethroids [2]. Triatominae control strategies were originally based on ecoepidemiological characteristics of the group, such as their slow reproduction and vulnerability to chemical control, and aimed to eliminate *T. infestans,* despite the fact that this species has restricted wild foci [3]. Thereby in the last decades, Uruguay, Chile and Brazil were certified as free of transmission of *T. cruzi* by *T. infestans* [4,5].

However, in the Gran Chaco in Argentina, Bolivia and Paraguay and in some areas of the Inter-Andean Valleys of Bolivia, despite the constant efforts to control *T. infestans* the success of these actions was limited and thus the species still persists [6,7]. In addition to this important problem, in the last few years, wild foci of *T. infestans* have been described, mainly in the Inter-Andean Valleys and in the Gran Chaco [8,9]. This fact was also observed in Argentina, Paraguay and Chile, showing that wild *T. infestans* have dispersed more widely than expected [10-14]. In Bolivia, the epidemiological significance of wild foci of *T. infestans* has been stressed [10].

The process of reinfestation of houses treated with insecticides is a serious phenomenon, and it is occurring quickly in the Gran Chaco [6,15-17]. It is considered that vector control failures, are due to high levels of insecticide resistance in this area [18-21]. In the last few years, more studies demonstrated that the phenomenon of resistance to insecticides in domestic *T. infestans* populations presents an extensive distribution in southern Bolivia and northern Argentina [21-23], with high resistance ratios, and different toxicological profiles [14,18,19,24-26]. Recently, susceptibility and resistance to deltamethrin and fipronil were detected in wild populations of *T. infestans* from Bolivia [14,27].

Coincidence or not, it is in those regions that the reach of the vector control strategies is limited, and insecticide resistance in *T. infestans* populations has been reported, including wild populations [14,27-29]. Thus, this study proposes to characterize the susceptibility to deltamethrin of wild and domestic *T. infestans* from the Gran Chaco and Inter-Andean Valleys in Bolivia, in order to understand better the extent of this relevant problem.

## Methods

### Populations of *T. infestans*

Nine populations of *T. infestans* five wild (S) and four domestic (D) were collected in the period from 2010 to 2011, in the region of Gran Chaco and Inter-Andean Valleys in Bolivia.

Wild *T. infestans* were captured using traps described by Noireau *et al.* [30,31]. In the Gran Chaco, they were captured in hollow tree trunks, whereas in the Inter-Andean Valleys in rock outcrops.

The collection of the domestic *T. infestans*, both in the Inter-Andean Valleys and in the Gran Chaco, was performed through active searches in intradomicile and peridomicile, with assistance of technicians from the National Chagas Disease Control Program of Bolivia (NCHDCP) (Table 1).

**Table 1:** **Samples of wild (S) and domestic (D) populations of** ***Triatoma infestans*** **, geographical origin, capture site (ecotope), morphs and cytogenetic classification**

**Site of collection**	**Department/Province**	**Latitude/longitude**	**Altitude meters**	**Capture site Ecotope**	**Morphs** ^**a**^	**Cytotype** ^**b**^
CIPEIN [SRL]	Susceptible reference strain	-	-	-	-	-
San Silvestre - S	Santa Cruz de la Sierra/Cordillera	19°21’21”S/62°34’10”W	400	Tree trunk	Dark morph	Non-andean
Terra Plem –S	Santa Cruz de la Sierra/Cordillera	19° 09’27”S/62°38’8”W	400	Tree trunk	Dark morph	Non-andean
Tita Chaco – S	Santa Cruz de la Sierra/Cordillera	18°55’39”S/62°34’28”W	400	Tree trunk	Dark morph	Non-andean
Mataral - S	Cochabamba/Aiquile	18°36’06”S/65°07’20”W	1,700	Rock outcrop	Mataral morph	Andean
Cotapachi – S	Cochabamba/Quillacollo	17°25’29”S/66°15’56”W	2,556	Rock outcrop	Common morph	Andean
Mataral - D	Cochabamba/Aiquile	18°35’44”S/65°08’58”W	1,750	Domestic (intra and peridomicile)	Common morph	Andean
Tamachindi - D	Santa Cruz de la Sierra/Cordillera	19°28’41”S/19°28’41”W	410	Domestic (intra and peridomicile)	Common morph	Non-andean
Rancho Nuevo –D	Santa Cruz de la Sierra/Cordillera	19°26’22”S/62°34’05”W	410	Domestic (intra and peridomicile)	Common morph	Non-andean
Villa Montes - D	Tarija/Gran Chaco	21°09’02”S/63°21’56”W	463	Domestic (peridomicile)	Common morph	Intermediate

^a^Morphs classification according to Noireau *et al*. [32], Cortez *et al*. [33].

^b^Cytotypes classification according to Panzera *et al*. [34].

All insects collected were identified using the taxonomic key of Lent and Wygodzinsky [35] and maintained under controlled conditions of temperature and humidity (25°C ±1°C; 60% ±10% RH). They were fed weekly with chicken blood (*Gallus gallus*), ethical approval Comissão de Ética no Uso de Animais (PROTOCOL N^o^ 41/14-2).

### Insecticide

Deltamethrin (pyrethroid) technical grade (S) – cyano-3-pehoxybenzyl (1R) -cis-3- (2,2-dibromovinyl) -2,2-dimethylcyclopropane Carboxylate, (99.6% - Bayer®, Brazil) was used.

### Bioassays

The susceptibility reference lineage (SRL) of *T. infestans* came from Centro de Investigaciones de Plagas e Insecticidas (CIPEIN) [36], preserved in the laboratory for more than 30 years, without contact with insecticide and inclusion of external material was used.

Serial dilutions of deltamethrin in acetone were prepared. For each concentration, three repetitions were carried out with ten first instar nymphs of F1 generation (five days old, fasting, weight of 1.2 ± 0.2 mg). The treatment consisted of the application of 0.2 μL of insecticide dilution on the dorsal abdomen, according to the World Health Organization- WHO [37] and Pessoa [38] procedures, with the aid of a Hamilton micro-syringe mounted on a repeating dispenser. For each population, a minimum of eight doses of insecticide active ingredient (a.i.) ranging from 0.42 to 300 ng and killing between >0% to <100% of the individuals, were applied per insect. Acetone was applied to the control group. The mortality was assessed 72 hours after application and it was determined by the inability or lack of coordination of the nymphs to move from the center to the edge of the filter paper (7 cm diameter). Signs of paralysis and lack of response to external stimuli was considered as well. During and after the experiment, the insects were kept under controlled conditions of temperature and humidity (25°C ±1°C; 60% ±10% RH).

### Diagnostic dose

The diagnostic dose (DD) applied was twice the minimum concentration of the insecticide that causes 99% of mortality in the susceptible laboratory strain [21,39]. According to the World Health Organization [39], when mortality is <80% the tested population is considered resistant, and if >98% it is considered as susceptible. The LD_99_ to deltamethrin of the SRL was determined (5.50 ng (a.i.) per insect) and with it the DD was estimated.

### Data analysis

Data from dose – response tests from each population were analyzed using the PROBIT program version 2 [40]. The slope and the lethal doses required to kill 50% of treated individuals (LD_50_) were estimated, as well as the Resistance Ratio (RR_50_), which was calculated by dividing each field population LD_50_ by the SRL LD_50_ value.

## Results

### Wild populations

From the five wild populations studied, the ones collected at the San Silvestre, Terra Plem and Tita Chaco communities (Chaco region) were identified as *T. infestans* dark morph. These populations had a RR_50_ lower or equal to the SRL, and 100% mortality to DD (Table 2). In agreement with PAHO criteria, they were considered as susceptible to deltamethrin since all had a RR <5.

**Table 2:** **Toxicological profile to deltamethrin in wild and domestic** ***Triatoma infestans*** **from Gran Chaco and the Inter-Andean Valleys of Bolivia**

**Population**	**N°**	**Slope +/− SD**	***X*** ^***2***^ ***(df) P***	**LD**_**50**_ **(95% CI)**	**RR**_**50**_ **(95%)**	**DD% (N°)**
CIPEIN (SRL)	240	2.83 +/−0.04	3.43 (4) 0.51	0.42 (0.35 - 0.49)	-	-
San Silvestre – S	300	1.97+/−0.05	0.51 (6) 0.00	0.26 (0.21 - 0.32)	0.62	100 (60)
Terra Plem – S	300	2.61 +/− 0.03	2.72 (6) 0.15	0.39 (0.33 - 0.46)	0.93	100 (60)
Tita Chaco – S	270	2.72+/−0.04	0.54 (5) 0.01	0.48 (0.41 - 0.58)	1.16	100 (60)
Mataral - S	390	4.36 +/− 0.02	1.75 (9) 5.16	1.78 (1.63 - 1.93)	4.24	96 (60)
Cotapachi – S	240	4.69+/−0.02	1.72 (6) 0.05	1.22 (1.11 -1.34)	2.90	100 (66)
Tamachindi - D	390	5.46 +/− 0.02	1.43 (9) 2.35	1.75 (1.62 - 1.87)	4.21	93(60)
Rancho Nuevo –D	390	4.43+/− 0.02	2.41 (10) 7.94	2.09 (1.93 - 2.27)	5.04	93 (45)
Mataral - D	480	2.92+/− 0.30	1.84 (2) 0.00	3.52 (3.15 - 4.02)	8.49	62 (60)
Villa Montes - D	360	2.25 +/− 0.04	3.05 (9) 0.04	54.23 (45.54 - 63.32)	129.12	0 (50)

SD: standard deviation; X^2^: chi-squared; *df*: degrees of freedom; P: probability value; LD_50_: Insecticide dose that killed 50% of the population (ng/insect); CI: confidence intervals; RR: resistance ratio; DD: % mortality of the discriminating dose; SRL: susceptible reference lineage; S: Sylvatic (wild); D: Domestic; N°: number of individuals used.

Regarding the populations from the Inter-Andean Valleys, *T. infestans* from the Cotapachi community were identified as common morph and presented a RR_50_ of 2.90 and 100% mortality to the DD. Furthermore, Mataral community *T. infestans* individuals were identified as Mataral morph and presented a RR_50_ of 4.24 and 96% mortality to the DD (Table 2).

Interestingly, we observed that *T. infestans* dark morph from the Chaco had lower slopes than the SRL (<2.83), whereas Mataral and Cotapachi populations had higher slopes (4.36 and 4.69, respectively).

### Domestic populations

Out of the four domestic populations studied, all bugs were identified as *T. infestans* common morph. Domestic populations from Mataral had a RR_50_ of 8.49 and 62% mortality to the DD. In the Rancho Nuevo and Tamachindi populations the RR_50_ was 4.21 and 5.04 respectively with 93% mortality to the DD for both communities. The level of resistance estimated for the Villa Montes population (RR_50_ = 129.12) and the mortality % to the DD (0%) drew our attention (Table 2).

Regarding the estimated slopes, the Tamachindi and Rancho Nuevo populations presented higher values than the SRL (5.46 and 4.43 respectively). On the other hand, resistant individuals from Mataral and Villa Montes presented slopes similar to the SRL (2.92 and 2.25 respectively).

## Discussion

This study shows the high susceptibility to deltamethrin determined for three wild populations of *T. infestans* dark morph from the Gran Chaco region of Bolivia, which in turn corresponds to the non-Andean region according to the cytogenetic classification of Panzera *et al.* [34]. These populations presented RR_50_ values equal to or less than the SRL. According to the criteria established by PAHO [41] they were considered susceptible to the tested insecticide (RR_50_ < 5). Notwithstanding, wild Andean populations from Mataral and Cotapachi were less susceptible than SRL and *T. infestans* dark morph populations.

Interestingly, wild *T. infestans* Mataral morph had a RR_50_ = 4.24 and thus they are less susceptible than the SRL, but less thanRR_50_ = 5 (PAHO criteria) and thus would be considered as susceptible. However, previous studies performed by Roca-Acevedo *et al.* [27] on the same region had reported individuals resistant to deltamethrin and fipronil (RR_50_ = 11.9 e 23.4 respectively). Additionally, in our study of domestic *T. infestans* in the same area we observed a deltamethrin RR_50_ of 8.49 whereas Roca-Acevedo *et al.* [27] reported a RR_50_ of 17.4. The differences between the values may be due to the fact that Roca-Acevedo *et al.* [27] used a different SRL to estimate the RR_50_.

Depickère *et al.* [14] reported 96% mortality to the deltamethrin DD in individuals from a wild Mataral population. Our results for individuals from the same population agree with this report. The same authors reported the susceptibility of eight wild populations of *T. infestans,* corresponding to the Andean region [34] from Bolivia. Among the populations they tested, the one from Cotapachi, presented a 100% mortality to the DD. Our study also evaluated a wild population from the same region, and similar qualitative test results were obtained (100% mortality to the DD). Nevertheless, the individuals we tested were less susceptible to deltamethirn than the SRL (RR_50_ = 2.90).

The DD is a qualitative method for rapid detection of resistant populations. Several *T. infestans* insecticide resistance studies have evaluated toxicological profiles using RR and DD criteria [14,18-21,27]. Nevertheless, the results obtained by both criteria are not always congruent. Thus, Picollo [42] proposed that the single dose killing 99% of the SRL (1X LD_99_) would be a more appropriate DD value. This dose permits detecting high mortality among susceptible individuals and low mortality among resistant. Increasing the DD value to twice the LD_99_, as proposed by Lardeux *et al.* [21], carries the risk of identifying resistant individuals as susceptible due to the high mortality % that would be estimated. This could occur mainly in populations that are in the process of selection for the resistance character, populations in which the toxicological profile would be masked. In contrast, populations with an established resistance character would not present this problem.

We consider that the sample number is an important limiting factor when assessing insecticide resistance. In addition, stochastic variability sources within the studied population must be taken into account. The study developed by Amelotti *et al.* [43] showed that females within an age range can produce individuals with different susceptibility profiles. Due to that, they recommended increasing the sample number to at least 60 individuals with no less than 10 females and with different ages represented per population. This approach would increase the reliability of the obtained results, avoiding false negatives and reducing incorrect interpretation.

During our study domestic populations of *T. infestans* from the Tamachindi and Rancho Nuevo communities, both from the Gran Chaco of the Santa Cruz Department (non-Andean region), were also evaluated. These populations were less susceptible than the SRL with a RR_50_ of 4.21 and 5.04 respectively. They both had 93% mortality to the DD. In several communities of the same region Depickère *et al.* [14] reported reduced susceptibility to deltamethrin in domestic *T. infestans*. It is possible that lower levels of insecticide susceptibility play an important role in the reinfestation process of domestic dwellings. Population genetics studies performed by Quisberth *et al*. [44] have confirmed that the reinfestation of houses in these communities happens due to residual populations and not due to invasion of wild bugs.

However, the resistance levels registered for populations collected in communities from the Gran Chaco of the Santa Cruz Department (non-Andean region) both in our study and by Depickère *et al.* [14], are not as high as the levels we registered for the Villa Montes population (RR >129.12 and 0% mortality to the DD). The latter, is considered as an Intermediate group, a result of the cross between Andean and non-Andean individuals [45]. Lardeux *et al.* [21] and Depickère *et al.* [14] emphasized that *T. infestans* populations from the Santa Cruz Department, where non-Andean populations occur, are more susceptible to deltamethrin. So Tarija populations (Intermediate), where high levels of resistance have been observed, also show low levels of mortality to the DD (<20%) [14,19-21]. These observations are very important since they correspond to a border area between Argentina and Bolivia, where high resistance levels have been reported [18,20].

Our study has also evaluated different *T. infestans* domestic and wild morph populations from different regions in Bolivia, both from the Inter-Andean Valleys (Andean region) and Gran Chaco (non-Andean region). For each population we obtained different susceptibility profiles. Recent studies indicate that most wild populations of *T. infestans* of the Andean regions in Bolivia are susceptible to deltamethrin [14,21,27]. However, resistance to deltamethrin and fipronil has also been reported in some wild populations from the Julo Grande and Kirus Mayu (Potosí Department) and Mataral (Cochabamba Department) in Bolivia [14,27]. Thus, our data and the aforementioned reports, support the idea that populations of *T. infestans* from different geographic areas and morphs have different toxicological profiles [15,21,22,27].

Population genetics studies consider Bolivia as a center of origin and dispersion of *T. infestans* [8,45-48]. The existence of wild foci and different morphs of this species, added to its wide distribution in that country [8-10,49], suggest a high genetic variability of this species in Bolivia. Therefore, Dias & Schofield [50] consider that the high genetic variability of *T. infestans* would explain why natural resistance and high levels of resistance to insecticides have developed in Bolivia.

Slope values have been used as indicators of population heterogeneity [27]. High slope values are related to low genetic variation, whereas populations in process of selection and thus showing genetic variation relate to less steep slopes (when compared to SRL slope) [51]. In this study 4 out of the 9 tested populations had values that suggest phenotypic variation. The three wild dark morph susceptible populations, and the domestic Villa Montes resistant population (Table 1).

The different toxicological profiles determined for domestic and wild populations of *T. infestans* could be the result of selective pressure from insecticide application plus the genetic variability of the highly structured populations present in this country [52-56]. Moreover, genetic studies through chromosomal markers performed by Panzera *et al.* [45] suggest that pyrethroid resistant populations from the Argentinean-Bolivian border are most likely the result of recent secondary contact between both chromosomal groups (Andean and non-Andean) suggesting a correlation between genomic variability and insecticide resistant populations.

The origin of resistance is unknown in wild Bolivian populations, because they have never had contact with insecticides and is probably due genetic variability. However, more genetic studies should be performed to characterize the resistance phenotype.

## Conclusion

Wild and domestic *T. infestans* populations from the Inter-Andean Valleys (Andean region) and Gran Chaco (non-Andean region) from Bolivia, have different susceptibility profiles towards deltamethrin. Although most wild populations are susceptible, insecticide resistance was observed in one. The existence of wild foci and different morphs of *T. infestans* plus its wide distribution in Bolivia are indicative of genetic variability. This could explain the occurrence of resistance in wild populations and thus we suggest that more genetic studies are performed on these populations and the resistance phenotypes are tested under field conditions.

## Abbreviations

Ng: Nano grams
mg: Milligrams
a.i: Active ingredient
°C: Celsius degree
μL: Microliters
RH: Humidity relative
